# 

**Published:** 1910-03

**Authors:** 


### BOOKS RECEIVED.

Modern Surgery. General and Operative. By John Chalmers DaCosta, M.D., Professor of Surgery and Clinical Surgery in Jefferson Medical College, Philadelphia. Sixth edition, revised and enlarged. Octavo, pp. 1502. With 966 illustrations, some in colors. Philadelphia and London. W. B. Saunders Co. 1910. (Cloth, $5.50.)

Anatomy and Physiology for Nurses. By LeRoy Lewis, M.D., Surgeon to and Lecturer on Anatomy and Physiology for Nurses at the Lewis Hospital, Bay City, Mich. Second edition. 12mo, pp. 344. Illustrated. Philadelphia and London: W. B. Saunders Co. 1910. (Cloth, $1.75.)

The Elements of the Science of Nutrition. By Graham Lusk, Ph.D., M.A., F.R.S. (Edin.), Professor of Physiology at Cornell Medical School, New York. Second edition, revised. Octavo of 402 pages, illustrated. Philadelphia and London: W. B. Saunders Company, 1910. (Cloth, $3.00 net.)

Transactions of the American Association of Genitourinary Surgeons. Vols. 3 and 4. Twenty-second and twenty-third annual meetings held at Hot Springs, Va., May 1 and 2, 1908, and at Mount Pocono, Pa., May 31, and June 1, 1909. Edward L. Keyes, Jr., Secretary.

Diagnostic Therapeutics. By Albert Abrams, A.M., M.D. (Heidelberg), Consulting Physician to the Mount Zion Hospital and the French Hospital, San Francisco. Octavo, pp. 1039. With 198 illustrations. New York: Rebman Co. (Cloth, $5.00.)

Nutrition and Dietetics. By Winfield S. Hall, Ph.D., M.D., Professor of Physiology in Northwestern University Medical School, Chicago. Small octavo, pp. 315. New York and London: D. Appleton & Co. 1910. (Cloth, $2.00.)

Medical Diagnosis. By Charles Lyman Greene, M.D., Professor of Medicine and Chief of the Department in the College of Medicine, University of Medicine, Minneapolis, Minn. Third edition. 12 mo, pp. 725. Illustrated. Philadelphia: P. Blakiston's Son & Co. (Leather, $3.50.)

Preparatory and After Treatment in Operative Cases. By Herman A. Haubold, M.D., Clinical Professor in Surgery and Demonstrator of Operative Surgery in New York University and Bellevue Hospital Medical College, New York. Octavo, pp. 650. With 429 illustrations. New York and London: D. Appleton & Co. 1910. (Cloth, $6.00.)

Diseases of the Genitourinary Organs. By Edward L. Keyes, Jr., M.D., Ph.D., Clinical Professor of Genitourinary Surgery in the New York Polyclinic Medical School. Octavo, pp. 975. With 195 illustrations in the text and 7 plates, four of which are colored. New York and London: D. Appleton & Co. 1910. (Cloth, $6.00.)

